# Clinical value of serology for the diagnosis of strongyloidiasis in travelers and migrants: A 4-year retrospective study using the Bordier IVD^®^ *Strongyloides ratti* ELISA assay

**DOI:** 10.1051/parasite/2021075

**Published:** 2021-12-06

**Authors:** Brice Autier, Sarrah Boukthir, Brigitte Degeilh, Sorya Belaz, Anne Dupuis, Sylviane Chevrier, Jean-Pierre Gangneux, Florence Robert-Gangneux

**Affiliations:** 1 Univ Rennes, CHU Rennes, Inserm, EHESP, Irset (Institut de Recherche en Santé Environnement Travail), UMR_S 1085 35000 Rennes France; 2 CHU Rennes, Laboratoire de parasitologie-mycologie 35000 Rennes France; 3 Univ Rennes, CHU Rennes, Inserm, CIC-1414 35000 Rennes France; 4 CHU Rennes, Laboratoire de bactériologie-hygiène hospitalière 35000 Rennes France

**Keywords:** Strongyloides, Serology, Diagnosis, Helminth

## Abstract

*Strongyloides stercoralis* serology is a sensitive method for strongyloidiasis diagnosis, but it is prone to cross-reactions with other helminthiases. This four-year retrospective study aimed at estimating the performance of the Bordier IVD^®^ *Strongyloides ratti* ELISA assay in a non-endemic country (France). The study included all patients tested for strongyloidiasis in our center between 2015 and 2019, by both serology and stool examination. Cases were defined using an algorithm considering serological results, microscopic examination of stools, and other biological, clinical or epidemiological data. The study included 805 stools from 341 patients (70% migrants, 20% travelers, 10% without travel to a highly endemic area). Thirty patients (8.8%) had positive serology, 9 had microscopically proven strongyloidiasis, and 11 and 10 were classified as probable and possible strongyloidiasis, respectively. Performances of microscopy and serology were compared, considering proven and probable strongyloidiasis as true infections. The sensitivity, specificity, positive predictive value and negative predictive value of serology were 100%, 97%, 67% and 100%, respectively, and those of microscopic examination of stools were 45% (*p* < 0.01), 100% (*p* < 0.01), 100% (*p* = 0.079) and 96% (*p* < 0.001), respectively. Eosinophilia did not help in discriminating true-positive from false-positive results. Overall, these results underline the high value of the *S. stercoralis* serologic assay, compared to stool examination. The systematic use of this technique for screening purposes in travelers or migrants, or before onset of immunosuppressive therapy, could help to improve patient management and epidemiological knowledge.

## Abstract (fr)

La sérologie de *Strongyloides stercoralis* est une méthode sensible pour le diagnostic de la strongyloïdose, mais elle est sujette à des réactions croisées avec d’autres helminthes. Cette étude rétrospective sur 4 ans visait à estimer les performances du test ELISA *Strongyloides ratti* Bordier IVD^®^ dans un pays non endémique (la France). L’étude a inclus tous les patients testés pour la strongyloïdose dans notre centre entre 2015 et 2019, à la fois par sérologie et examen des selles. La définition des cas a été faite à l’aide d’un algorithme tenant compte des résultats sérologiques, de l’examen microscopique des selles et d’autres données biologiques, cliniques ou épidémiologiques. L’étude a inclus 805 selles de 341 patients (70 % de migrants, 20 % de voyageurs, 10 % sans voyage dans une zone de forte endémie). Trente patients (8,8 %) avaient une sérologie positive, 9 avaient une strongyloïdose prouvée au microscope, et 11 et 10 ont été classés respectivement comme strongyloïdose probable et possible. Les performances de la microscopie et de la sérologie ont été comparées, en considérant les strongyloïdoses avérées et probables comme de véritables infections. La sensibilité, la spécificité, la valeur prédictive positive et la valeur prédictive négative de la sérologie étaient de 100 %, 97 %, 67 % et 100 %, respectivement, et celles de l’examen microscopique des selles étaient de 45 % (*p* < 0,01), 100 % (*p* < 0,01), 100 % (*p* = 0,079) et 96 % (*p* < 0,001), respectivement. L’éosinophilie n’a pas aidé à distinguer les vrais positifs des faux positifs. Dans l’ensemble, ces résultats soulignent la valeur élevée du test sérologique de *S. stercoralis*, par rapport à l’examen des selles. L’utilisation systématique de cette technique à des fins de dépistage chez les voyageurs ou les migrants, ou avant le début d’un traitement immunosuppresseur, pourrait contribuer à améliorer la prise en charge des patients et les connaissances épidémiologiques.

## Introduction

Strongyloidiasis is a soil-transmitted helminthiasis (STH) caused by *Strongyloides stercoralis*, a roundworm parasite of humans. Its complex life cycle includes an endogenous cycle of reinfection, allowing the persistence of the parasite, even decades after having left an endemic area [22]. It has been estimated that 600 million people worldwide are infected with *S. stercoralis* [8], while the true prevalence could be higher, due to the challenging diagnosis and the frequency of asymptomatic carriage. Strongyloidiasis is preferentially distributed in low-income tropical and sub-tropical countries, because of specific hygroscopic and thermic requirements for larvae survival in the environment, and because of the trans-cutaneous mode of contamination through barefoot walking on feces-contaminated soil. In non-endemic countries, strongyloidiasis is also frequently diagnosed in travelers to or migrants from endemic areas [30], but autochthonous infections remain possible in areas with favorable conditions [7].

*Strongyloides stercoralis* is responsible for a broad spectrum of clinical presentations, including digestive symptoms (diarrhea, epigastric pain) possibly associated with cutaneous and/or respiratory signs (*larva currens*, erythematous rash, dry cough, asthma). Many patients with chronic strongyloidiasis are asymptomatic as long as they are immunocompetent [18], due to the particular nature of the endogenous parasite life cycle ensuring silent self-persistence. Some patients, often with mild immunosuppression, may present hyperinfection with exacerbated clinical signs. In cases of deeper immune deficiency, infection can evolve to life-threatening disseminated strongyloidiasis [1, 10]. The severity of disseminated strongyloidiasis is high, reaching a mortality rate up to 70% [10], making it essential to treat *Strongyloides* carriers before immune suppression [14, 23].

Diagnosis of *S. stercoralis* infection usually relies on stool examination using specific techniques, but microscopic diagnosis is challenging due to the intermittent and low-number shedding of larvae in stools. It relies either on the hygrotropism and thermotropism of the rhabditoid larvae released by adult females in the gut (Baermann concentration), or on *in vitro* stool culture at 27 °C, mimicking the external life cycle of the parasite (Harada-Mori filter paper culture, Arakaki agar culture), thus requiring fresh stools containing live larvae. Even with these specific techniques, microscopy is known to have poor sensitivity; therefore, multiple samples are needed to improve performances of stool examination and counterbalance intermittent shedding [28]. However, it may be difficult for clinical laboratories to obtain multiple freshly emitted stools, notably from patients who do not comply for cultural reasons. By contrast, antibody levels are steady as long as infection persists. As a result, serology is considered to be a valuable tool for patient screening.

Various serological techniques have been described, either in-house (immunofluorescence, agglutination, western-blot, ELISA), or marketed (ELISA) methods, but their performances vary according to the type of antigen used and the method used as a reference [24]. As serology is reputed to be more sensitive than microscopy, it has been proposed as a screening tool before immune suppression and/or in migrants [11, 23, 25]. Moreover, serology can contribute to diagnosis of newly acquired infections, as antibodies serorevert within 12–18 months of successful treatment [16]. However, cross-reactions are common in case of infection with other nematodes, especially those with tissue larval migration, such as filariasis and toxocariasis, the prevalence of which may differ depending on the country or the population studied [24].

In the present study, we retrospectively evaluated the diagnostic efficacy of stool microscopic examination and serology in a four-year cohort, using the Bordier IVD^®^ *Strongyloides ratti* ELISA assay (Bordier Affinity Products, Crissier, Switzerland), to determine the performance of this assay for screening purposes in a non-endemic area.

## Materials and methods

### Ethics approval

The study was submitted to the Ethics Committee of the Rennes University Hospital, and received approval No. 20.09.

### Patients and samples

All patients who were prescribed specific examinations for strongyloidiasis in our hospital from November 2015 to November 2019, and who benefited from both microscopic examination of stools for *S. stercoralis* and serology, were included in the study. Serology was considered concomitant to stool examination when blood was sampled within a maximum one-month interval from stool sample.

Patients born in a non-endemic area were considered travelers if they had been in an area known to be highly endemic for strongyloidiasis irrespective of the time period [19], and as migrants if they were born in an endemic area. They were prescribed examination for strongyloidiasis, either because of suggestive clinical signs, or to investigate hypereosinophilia, or before initiation of immunosuppressive therapy. Clinical and epidemiological data were collected by retrospective examination of medical charts and consultation reports.

### Serology

*Strongyloides* serology was performed using the commercial Bordier IVD^®^ *Strongyloides ratti* ELISA assay (Bordier Affinity Products, Crissier, Switzerland) following the manufacturer’s instructions, within 7 days after blood sampling. The result was expressed as an ELISA index defined as the ratio of absorbance obtained with the sample/absorbance obtained with the threshold control. Index results above 1.0 and under 0.9 were considered positive and negative results, respectively. An index result ranging from 0.9 to 1.0 was considered as “doubtful”, and a control sample was requested 2–4 weeks later. In the absence of a control sample or persistence of an index between 0.9 and 1.0, serology was considered negative.

Toxocariasis and filariasis serological results were also taken into account to classify the cases, when available, and were obtained using the commercial Ridascreen^®^ *Toxocara* IgG (R-Biopharm, Darmstadt, Germany) and Bordier^®^ *Acanthocheilonema viteae* (Bordier Affinity Products, Crissier, Switzerland) ELISA assays, respectively, following the manufacturers’ recommendations. For samples positive for *Toxocara* ELISA, a confirmatory test using the *Toxocara* Western Blot IgG assay (LDBio Diagnostics, Lyon, France) was performed.

### Stool examination

Baermann concentration and/or Harada-Mori filter paper culture were undertaken immediately after sample reception [17]. Techniques were performed by trained operators on freshly emitted samples. The final result of the Harada-Mori technique was delivered after 10 days of culture at 25 °C. For both the Baermann and Harada-Mori methods, the whole pellet obtained after centrifugation of the saline aqueous phase was read after wet mount. Also, for all samples, direct wet mounts were performed on fresh stool and after concentration methods (Bailenger’s, Thebault’s and/or Merthiolate-Iodine-Formalin biphasic methods), as routinely done in our laboratory for every microscopic examination of stools for ova and parasites.

### Case definition

In the absence of a gold standard, case definition was graduated following an algorithm, taking into account stool examination and serological results, based on complementary data. Patients with negative serology and negative examination of stools were considered uninfected, and those with positive serology and positive examination of stools were classified as proven strongyloidiasis.

When serology was positive and stool examination was negative, patients were classified into two groups (probable strongyloidiasis and possible strongyloidiasis), after thorough review of medical charts, taking into account the clinical and epidemiological setting, alternative nematode diagnoses by stool examination, and the results of serology for other nematodes when available (toxocariasis, filariasis, anisakidosis, trichinellosis or other, depending on the context). Criteria were gathered in four grades, namely A–D, as described in Table 1.

**Table 1:** Criteria used for the classification of cases.

Grade	Description	Status
A	Presence of *S. stercoralis* larvae in stools	Proven strongyloidiasis
B	*S. stercoralis* serology index ≥ 1.5 and serologic results for other nematodes (i) negative or (ii) positive with a lower index than that of *S. stercoralis* serology^1^	Probable strongyloidiasis
C	*S. stercoralis* serology positive but patients not fulfilling grades A or B	Possible strongyloidiasis
D	*S. stercoralis* serology negative and absence of larvae detection	No strongyloidiasis

1 Direct comparison of serology indexes is possible only if both techniques have the same positivity threshold.

### Statistical analysis

Statistical tests and figures were done using GraphPad Prism v6. Sensitivities and specificities were compared using McNemar’s test; negative predictive values (NPV) and positive predictive values (PPV) were compared using Fisher’s exact test; eosinophil counts were compared using the Kruskal-Wallis test with a *post hoc* Dunn’s multiple comparison test; proportions of patients with eosinophilia were compared with Fisher’s exact tests and correlation between serology index; and eosinophil counts were tested with Spearman’s coefficient test. A *p*-value < 0.05 was considered statistically significant (*α* risk of 0.05).

## Results

### Patient characteristics

**Figure 1:**
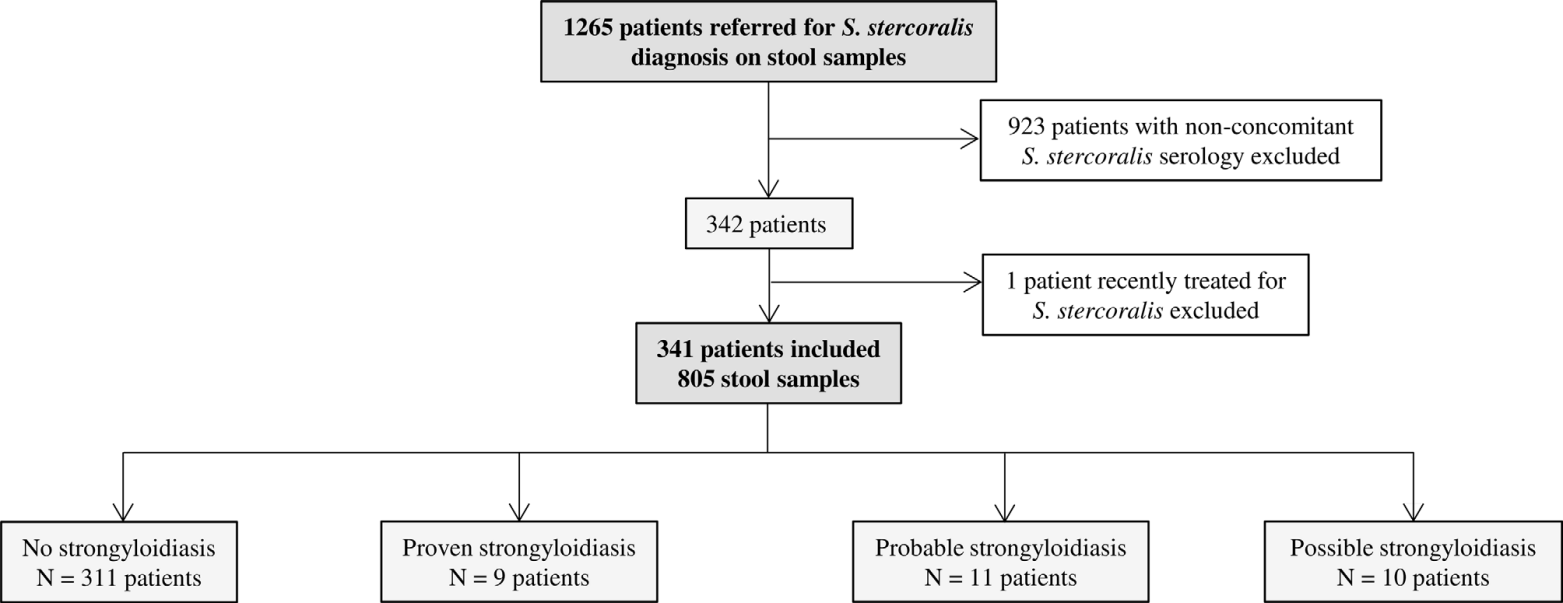
Flowchart of included cases.

From November 2015 to November 2019, 1265 patients benefited from a microscopic examination of stools for *S. stercoralis*. Among them, 923 patients had no concomitant serology and were excluded. One patient among the 342 remaining was investigated in the setting of post-treatment follow-up and was excluded (Fig. 1). For the 341 remaining patients, 805 examinations of stools for *S. stercoralis* were recorded: 619 with filter paper culture only, 118 with Baermann concentration only, and 68 with both techniques (Table 2). The number of stools analyzed for each patient ranged from 1 to 4 (median = 3). The mean age of the 341 patients was 33 years, and the male/female ratio was 2.2. Patients from the cohort were mainly migrants (70%, 240/341), but also travelers (20%, 68/341) or autochthonous patients with no history of travel to highly endemic areas (10%, 33/341). For most of them (90%, 306/341), the eosinophil count was available, and was most often lower than 0.5 G/L, i.e. considered normal (62%, 191/306). Patients with proven or probable strongyloidiasis received a single oral dose of ivermectin.

**Table 2:** Characteristics of the cohort (*N* = 341).

Demographic and clinical characteristics	Values
Male/female sex ratio	2.2
Age, mean ± *SD*	33 ± 17
Type of patient, % (*n*/*N*)	
Migrants	70% (240/341)
Travelers	20% (68/341)
Autochthonous	10% (33/341)
Hospital unit, % (*n*/*N*)	
Consultation of parasitology or infectious diseases	82% (279/341)
Immunocompromized patients	3% (10/341)
Other	15% (52/341)
Microscopic examination of stools, *N*	805
Number of samples/patient, median	3
% with 1 stool examination	21.1%
% with 2 stools examinations	24.9%
% with ≥ 3 stools examinations	54.0%
Concentration methods	
% of patients with ≥ 1 Harada-Mori filter paper culture (*N* stools)	98.5% (687)
% of patients with ≥ 1 Baermann concentration (*N* stools)	46.9% (186)
Number of patients with available eosinophil count (EC)	306
% with EC < 0.5 G/L	62.4%
% with EC [0.5; 1] G/L	16.1%
% with EC ≥ 1 G/L	21.3%

### Performance of diagnostic tests

All cases were classified following the algorithm detailed in Table 1. Among the 341 patients, 311 had no strongyloidiasis, while 9, 11, and 10 were classified as proven, probable and possible strongyloidiasis, respectively (Fig. 1). Among patients with positive serology, age, patient type, number of stool samples and microscopic methods used did not differ between microscopically positive and negative groups (Table 3). Overall, 23 stool samples were collected from patients with proven strongyloidiasis, of which 21 (91%) were microscopically positive for *S. stercoralis*. To estimate the performance of serology, patients with proven or probable strongyloidiasis were considered infected, whereas patients with possible strongyloidiasis were considered non-infected. Using this definition, the cohort consisted of 20 cases with strongyloidiasis and 321 patients without strongyloidiasis. The PPV and sensitivity for serology were 67% and 100%, respectively, while for stool examination they reached 100% and 45%, respectively (Table 4). NPV, sensitivity and specificity were significantly different between serology and microscopy (*p* < 0.001, *p* < 0.01 and *p* < 0.01, respectively), whereas no statistical difference was observed for PPV (*p* = 0.079). Among patients with possible strongyloidiasis, 7/10 were infected with another nematode (2 with *Loa loa*, 1 with both *Mansonella perstans* and an unidentified Ancylostomatidae, 2 with *Toxocara* sp., 1 with *Trichuris trichiura* and 1 with *Enterobius vermicularis*), which could account for cross-reactions (Table 5).

**Table 3:** Detailed characteristics of patients with a positive serology.

	Patients with positive serology and		Statistical significance
	Microscopy negative	Microscopy positive	
	*N* = 21	*N* = 9	
Demographic characteristics			
Age, mean ± *SD*	35 ± 19	41 ± 18	ns
% of migrants	71% (15/21)	78% (7/9)	ns
Stool examination for *Strongyloides* larvae			
% of patients with ≥ 3 stool examinations (*n*/*N*)	52% (11/21)	56% (5/9)	ns
% of patients with ≥ 1 Harada-Mori filter paper culture (*n*/*N*)	100% (21/21)	100% (9/9)	ns
% of patients with ≥ 1 Baermann concentration (*n*/*N*)	48% (10/21)	67% (6/9)	ns
Eosinophil counts (G/L, median (interquartile range))	0.47 (0.05;12.18)	1.32 (0.57;2.18)	*

ns: not significant, *p < 0.05.

**Table 4:** Performance of serology and microscopic examination of stools for the diagnosis of strongyloidiasis (*N* = 20 proven/probable and 321 negative/possible cases).

	Microscopic examination of stools		*S. stercoralis* serology		Statistical significance
	% (*n*/*N*)	95% CI	% (*n*/*N*)	95% CI	
Sensitivity	45 (9/20)	26–66	100 (20/20)	81–100	**
Specificity	100 (321/321)	99–100	97 (311/321)	94–98	**
PPV	100 (9/9)	66–100	67 (20/30)	49–81	ns
NPV	97 (321/332)	94–98	100 (311/311)	99–100	***

PPV: positive predictive value, NPV: negative predictive value, ns: not significant, **p < 0.01, ***p < 0.001 (Fisher’s exact test).

**Table 5:** Detailed characteristics of patients with positive serology.

Case No.	Gender-Age (years)	Migrant/Traveler	Travels	*S. stercoralis* serology index	Other serologies (index or result/threshold)			Microscopic examination of stools (no. of stools)	Eosinophil count (G/L)	Clinical data	Final classification
					Filariasis	Toxocariasis	Other				
1	M-48	Traveler	Madagascar, French Guiana	4.6	nd	nd	–	*S. stercoralis* (3)	1.5	*Larva currens*	Proven
2	M-24	Migrant	Ethiopia, Sudan, Libya, Italy	2.7	Positive (2.0)	Positive (1.0)	–	*S. stercoralis* (4)	1.1	Asymptomatic	Proven
3	M-32	Migrant	Gabonese Republic	3.7	nd	nd	–	*S. stercoralis* (3)	0.8	Abdominal pain	Proven
4	F-19	Migrant	Mayotte	4.2	nd	nd	–	*S. stercoralis, Trichuris trichiura* (3)	1.7	Occasional diarrhea	Proven
5	M-35	Migrant	Ethiopia	3.4	Positive (3.0)	Negative	Ascaridiosis, trichinellosis (negative)	*S. stercoralis* (3)	0.9	HIV infection, gastric pain	Proven
6	M-35	Migrant	Cameroon, Benin	2.9	Positive (2.6)	Positive (1.1)	Ascaridiosis (negative)	*S. stercoralis, Necator americanus* (1)	1.3	Proven mansonellosis, fever	Proven
7	F-40	Traveler	Thailand	1.1	Positive (1.7)	Negative	Ascaridiosis, trichinellosis (negative), angiostrongylosis (positive WB)	*S. stercoralis* (2)	1.9	Epigastric pain, acute myocarditis	Proven
8	M-80	Migrant	Martinique Island	4.1	Positive (1.1)	Negative	–	*S. stercoralis* (2)	2.2	Diarrhea, weight loss	Proven
9	M-54	Migrant	Reunion Island	2.1	nd	nd	–	*S. stercoralis* (2)	0.6	Intermittent abdominal pain	Proven
10	F-47	Migrant	Guinea	2.9	Negative	Negative	Ascaridiosis, trichinellosis (negative)	Negative (2)	1.20	Chronic coughing and fever	Probable
11	M-25	Migrant	Ethiopia	2.5	Negative	Negative	–	*S. mansoni* (3)	0.50	Digestive discomfort	Probable
12	M-23	Migrant	Sudan	2.5	Negative	Negative	–	Negative (1)	0.05	Abdominal pain	Probable
13	M-16	Migrant	Guinea	2.4	Negative	Negative	–	Negative (1)	0.43	Abdominal pain, digestive bleeding	Probable
14	M-35	Migrant	Afghanistan	2.7	Positive (1.9)	Negative	–	Negative (2)	0.24	Diarrhea, digestive discomfort, epigastric pain	Probable
15	M-13	Migrant	Guinea	3.5	Positive (1.5)	Negative	–	*S. mansoni* (3)	0.50	Abdominal pain, diarrhea, itching	Probable
16	M-36	Traveler	Mali	1.7	Negative	Negative	–	Negative (1)	1.26	Dyspnea	Probable
17	M-48	Traveler	Gabonese Republic, Cameroon	3.5	Positive (3.3)	nd	–	Negative (3)	2.85	Tenesmus, loaosis	Probable
18	F-35	Migrant	Polynesia	1.6	Positive (1.3)	nd	–	Negative (1)	0.24	Fever under azathioprine treatment	Probable
19	F-67	Traveler	Cameroon, Republic of Cabo Verde	1.5	Negative	nd	Ascaridiosis (negative)	Negative (3)	0.14	Coughing, constipation	Probable
20	M-25	Migrant	Guinea	1.7	Negative	Negative	–	Negative (2)	nd	Meteorism, bloating	Probable
21	M-28	Migrant	Democratic Republic of Congo	1.4	nd	nd	–	Negative (3)	0.24	Epigastric pain	Possible
22	M-24	Migrant	Sudan	1.0	nd	nd	–	Negative (3)	1.77	Abdominal pain, diarrhea	Possible
23	F-5	Migrant	Romania	2.1	Negative	Positive (13.1)	Ascaridiosis (positive IEP)	Ancylostomatidae Eggs (3)	12.18	Geophagy, toxocariasis	Possible
24	M-27	Traveler	Democratic Republic of Congo	2.8	Positive (21.7)	Negative	–	Ancylostomatidae Eggs (2)	0.79	Proven mansonellosis	Possible
25	F-47	Traveler	Tunisia, Morocco	1.3	nd	Negative	Trichinellosis (negative)	*Enterobius vermicularis* (3)	0.44	Abdominal pain, fatigue	Possible
26	M-15	Migrant	Guinea, Senegal	1.3	Positive (1.6)	Negative	–	*Trichuris trichiura* (4)	0.09	Abdominal pain, constipation	Possible
27	M-77	Migrant	Cameroon	2.8	Positive (3.4)	Negative	Trichinellosis (negative)	Negative (3)	0.86	Clinical loaosis	Possible
28	F-66	Migrant	Cameroon	1.2	Positive (1.9)	nd	–	Negative (1)	nd	Proven loaosis	Possible
29	F-53	Traveler	Haiti	1.9	nd	Positive (3.3)	Anisakidosis (negative)	Negative (3)	nd	Toxocariasis	Possible
30	F-23	Traveler	Peru	1.1	nd	Negative	Ascaridiosis (negative)	Negative (2)	0.18	Diarrhea, abdominal pain	Possible
							Anisakidosis (0.59/0.40)				

nd: not determined; WB: Western-Blot; IEP: Immunoelectrophoresis.

### Diagnostic value of eosinophil count

**Figure 2:**
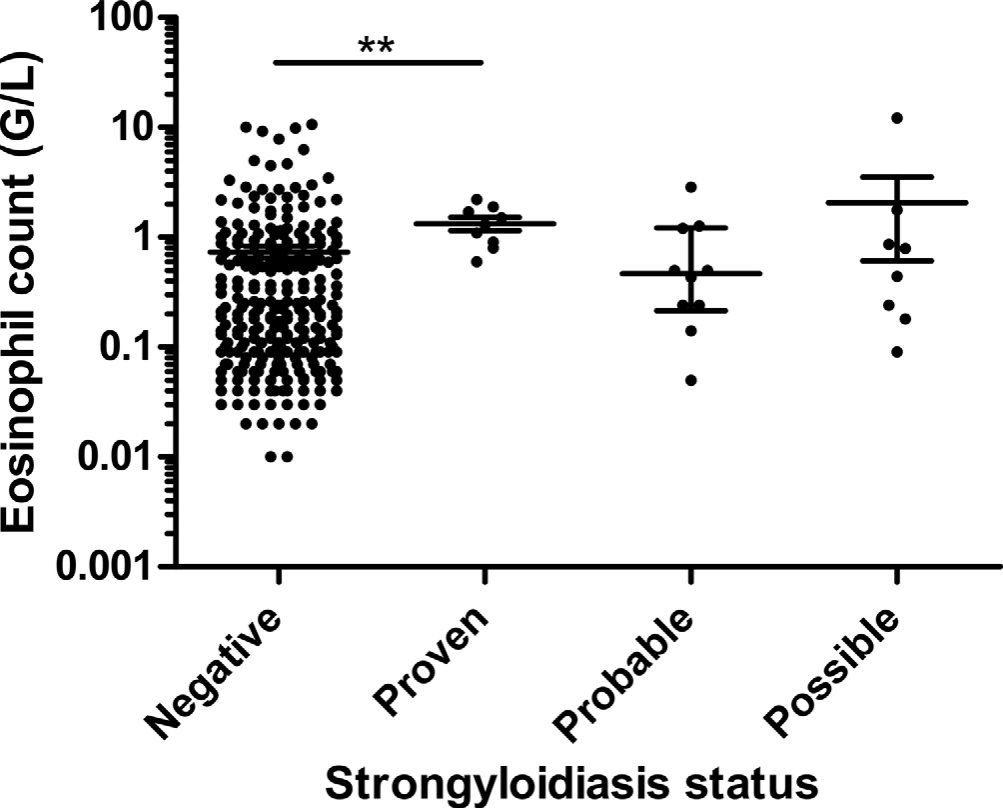
Eosinophil counts related to patient status. Median and interquartile range, **p < 0.01.

For 306 patients, the eosinophil count was available. The median eosinophil counts for proven, probable, possible strongyloidiasis and uninfected groups were 1.300, 0.465, 0.615 and 0.210 G/L, respectively (Fig. 2), with a statistically significant difference between the groups of uninfected patients and proven strongyloidiasis (*p* < 0.01) (Dunn’s Multiple Comparison Test). The proportion of patients with hypereosinophilia for the proven, probable, possible strongyloidiasis and uninfected groups were 100% (9/9), 50% (5/10), 50% (4/8) and 35% (97/279), respectively (*p* < 0.001). Using the previous division in infected/non-infected groups, hypereosinophilia was observed in 74% (14/19) of patients with strongyloidiasis and 35% (101/287) of patients without strongyloidiasis (significant, *p* < 0.01). Serological index and eosinophil count were weakly correlated, whether applying the test to all patients (Spearman’s *r* = 0.387, *p* < 0.05) or on strongyloidiasis cases only (Spearman’s *r* = 0.474, *p* < 0.05). In order to evaluate whether eosinophilia could help to interpret positive *S. stercoralis* serology, PPV and NPV of hypereosinophilia were calculated, considering only patients with positive serology, and reached 78% (14/18) and 44% (4/9), respectively.

## Discussion

In this 4-year retrospective study, we compared serology and stool examination for the diagnosis of *Strongyloides stercoralis* infection, in 341 patients tested in our tertiary care hospital. To date, there is no gold standard for strongyloidiasis diagnosis, as serological techniques are reputed sensitive but are prone to cross-reactions with other roundworms, and microscopic examinations lacks sensitivity [6]. Therefore, our classification of cases considered the results of both analyses. Additional clinical and epidemiological data were also collected to support this classification. As expected, serology had a much higher sensitivity than microscopy (100% versus 45%), meaning that it can be used for screening purposes with a theoretical 100% NPV. However, the PPV of 67% requires us to add other investigations to the workup, such as microscopic examination of stools for ova and parasites and serological tests for other nematodes. This strategy allowed us to discriminate 70% (7/10) of the putative false-positive results of serology.

We observed 7 putative cases of cross-reactions with other nematodosis, i.e. toxocariasis, filariasis, enterobiosis and whipworm infection (patients #23–29). As these infections are due to roundworms, they are highly susceptible to induce false positivity of the *S. ratti* ELISA assay. Therefore, in the case of hypereosinophilia, strongyloidiasis serology should never be an isolated test, otherwise the patient could be misdiagnosed as having strongyloidiasis. Although previous studies have reported more specific assays using recombinant *S. stercoralis* antigens, we still have to wait for the era of highly specific marketed assays [20, 21]. On the other hand, it should be kept in mind that strongyloidiasis can never be formally excluded, even in presence of an obvious source of cross-reaction, all the more so as parasitic coinfections are frequent in endemic areas. For this reason, the putative cross-reactions were still considered “possible” strongyloidiasis in this study. As an illustration, during another study involving some of these patients, we observed that one of the “possible strongyloidiasis” cases was in fact probably infected with *S. stercoralis* (patient #24), as highlighted by positive PCR assay on stool sample.

Eosinophil counts were significantly higher in the strongyloidiasis group compared to uninfected patients, but were not useful to distinguish probable strongyloidiasis from possible strongyloidiasis. In their recent work, Ming and colleagues observed normal eosinophil counts in 23% of their proven cases [18], which is close to our data (28% in proven + probable strongyloidiasis). This finding, together with the poor NPV and PPV values of eosinophil counts for patients with positive strongyloidiasis serology, confirms the low value of eosinophilia for strongyloidiasis diagnosis, as underlined in previous reports [14]. This is probably due to the particular pathophysiology of this infection, with possible prolonged carriage of female worms during endogenous cycles in asymptomatic patients. Other parameters associated with eosinophil activation, such as eosinophil cationic protein (ECP) or eosinophil peroxidase (EPO), could be of higher interested in the near future, as recently highlighted for solid organ transplant patients [12].

In our study, we obtained better performance for serology than previously reported. In their review, Requena-Méndez and colleagues highlighted that the sensitivity of ELISA assays for strongyloidiasis diagnosis ranged from 73% to 100% [24]. They suggested lower performances of the assays due to defective antibody production in immunocompromized patients [15, 28]. Our results were not affected by this limitation, as only one case of strongyloidiasis was diagnosed in an immunocompromized patient (HIV patient #5), who had strongly positive serology. In 2014, a comparative evaluation of five *S. stercoralis* serology assays, including that used in our study, was performed. The best sensitivity was obtained with an in-house IFAT assay (95%), but it also had the lowest specificity (83%), whereas the Bordier IVD^®^ ELISA assay showed sensitivity of 91% and specificity of 94% [4]. This evaluation was based on a composite cohort from the United States and Europe, with 114 microscopically proven infections, 16 putative cases without larvae detection in stools, and 269 patients who were not infected. Proportions of migrants and immunocompromized patients were not described in this study. More recently, a retrospective study on imported strongyloidiasis diagnosed in a tertiary care hospital from Western Europe (Hospital for Tropical Disease, London) [18] using the Bordier IVD^®^ assay showed sensitivity of serology of 81%, with a much lower performance in travelers (42%, 6/13) than in migrants (90%, 61/68). Sensitivity in the migrant group was similar to ours, and the overall poorer sensitivity could be due to the higher proportion of immunocompromized patients (23% versus 11% in our study), or to the time of sampling after contamination. Clearly, if serological testing occurs too early after contamination, the result might be negative, as IgG titers usually increase concomitantly to larvae detection in stools. This is less pivotal for other helminths such as schistosomiasis or fascioliasis [13, 27]. All positive cases in our cohort were diagnosed at least 40 days after the putative exposure, which could explain the perfect agreement of serology and stool examination compared to other published data [18]. Altogether, even though we observed perfect sensitivity of the Bordier assay in our study, others reported occurrences of false-negative results that prevent us from relying on it for screening before onset of immunosuppressive therapy, as highlighted by a recent cost-effectiveness study [29].

Recently, PCR assays have been developed for *S. stercoralis* detection in stools, often yielding better performances than microscopy [3, 5, 26]. However, these assays showed heterogeneous performances, greatly depending on the patient population and the reference method used for clinical evaluation [2, 9]. In a recent meta-analysis, Buonfrate et al. estimated the sensitivity of real-time PCR at 57%, compared to serological and/or parasitological methods, and 64%, compared to parasitological methods only [9]. Therefore, it should be kept in mind that PCR assays need further clinical evaluations before routine use in non-endemic countries. While PCR assays have great value due to high specificity, a negative result cannot exclude strongyloidiasis.

The limitations of our study are mainly the retrospective design and the size of the cohort. However, the thorough analysis of clinical and epidemiological data allowed us to provide a fair estimation of the status of patients regarding strongyloidiasis. Also, our cohort contained few immunocompromized patients, and the performance of the assay should be confirmed in this population. Finally, our results should not be extrapolated to other serological tests, which could use other antigens or other epitopes, and which could therefore perform differently.

Overall, high sensitivity and NPV support the use of the Bordier IVD^®^ ELISA assay for screening and diagnostic purposes, allowing better case estimation and detection of treatment failures. However, it should be associated with other serologic assays in order to highlight possible cross-reactions.

## Conflict of interest

The authors have declared that they have no conflict of interest.
